# A clinical study of the correlation between metabolic-associated fatty liver disease and coronary plaque pattern

**DOI:** 10.1038/s41598-023-34462-8

**Published:** 2023-05-04

**Authors:** Zhijiao Zhang, Mengyao Zheng, Hongtao Lei, Zimeng Jiang, Yuhang Chen, Haiyu He, Gongfang Zhao, Hua Huang

**Affiliations:** Department of Gastroenterology, The Second Affiliated Hospital of Kunming Medical University, No. 374, Dianmian Avenue, Wuhua District, Kunming City, 650000 China

**Keywords:** Hepatology, Gastroenterology

## Abstract

Nonalcoholic fatty liver disease (NAFLD) is the hepatic manifestation of metabolic syndrome (MetS) and has been correlated with coronary atherosclerosis (CAS). Since NAFLD was renamed metabolic-associated fatty liver disease(MAFLD) in 2020, no studies have evaluated the correlation between MAFLD and CAS. The aim of this study was to evaluate the relationship between MAFLD and CAS. A total of 1330 patients underwent continuous coronary computed tomography angiography (CCTA) and abdominal ultrasound as part of a routine physical examination. Ultrasonography was used to assess fatty liver, and CCTA was used to assess coronary artery plaques, degree of stenosis, and diseased blood vessels. Univariate and multivariate logistic regression analyses were performed with plaque type and degree of stenosis as dependent variables and MAFLD and traditional cardiovascular risk factors as independent variables to analyze the correlation between MAFLD and CAS. Among the 1164 patients, 680 (58.4%) were diagnosed with MAFLD through a combination of ultrasound and auxiliary examinations. Compared with the non-MAFLD group, the MAFLD group had more cardiovascular risk factors,and the MAFLD group had more likely to have coronary atherosclerosis, coronary stenosis and multiple coronary artery stenosis.In the univariate logistic regression, MAFLD was significantly correlated with overall plaque, calcified plaques, noncalcified plaques, mixed plaques,and significant stenosis in the coronary arteries.(*p* < 0.05). After adjusting for cardiovascular risk factors , MAFLD was correlated with noncalcified plaques (1.67; 95% confidence interval (CI) 1.15–2.43; *p* = 0.007) and mixed plaques (1.54; 95% CI 1.10–2.16; *p* = 0.011). In this study, MAFLD group had more cardiovascular risk factors, MAFLD was correlated with coronary atherosclerosis,and significant stenosis.Further study found independent associations between MAFLD and noncalcified plaques and mixed plaques, which suggest a clinically relevant link between MAFLD and coronary atherosclerosis.

## Introduction

As living standards have improved, the incidence of metabolic syndrome (MetS, including obesity and type 2 diabetes) is increasing annually. Metabolic-associated fatty liver disease (MAFLD), once known as nonalcoholic fatty liver disease (NAFLD), is the main cause of chronic liver disease. The prevalence of NAFLD in China was 32.9% in 2018^1^, and by 2030 the total number of NAFLD patients in China is expected to increase to 314.58 million, which would be the fastest increase in the prevalence of NAFLD in the world. Liver complications of NAFLD include nonalcoholic steatohepatitis (NASH), liver cirrhosis, and hepatocellular carcinoma. Although NAFLD is complicated with NASH in 10–25% of NAFLD patients, which can lead to liver cirrhosis, hepatocellular carcinoma and liver failure^2^, the main cause of death in NAFLD patients is cardiovascular disease (CVD)^3^. A variety of cardiovascular risk factors, such as hypertension, diabetes, obesity, and MetS, are usually present in NAFLD patients. NAFLD is closely correlated with atherosclerosis (AS)^4^, and it may have the same pathogenesis as CVD.

In 2020, an international panel of experts from 22 countries proposed a new definition of NAFLD^5^ and renamed this disease MAFLD. The diagnostic criteria for MAFLD are based on evidence of liver steatosis in patients with metabolic abnormalities (not related to alcohol consumption or the presence of comorbidities such as chronic viral hepatitis). However, because not all patients with NAFLD can be diagnosed with MAFLD and vice versa, the two terms are not interchangeable (kappa coefficient is 0.92)^6^. Since the renaming of NAFLD as MAFLD, studies have revealed that the correlation between MAFLD and CVD is stronger than that between NAFLD and CVD^7,8^. We conducted this retrospective cross-sectional study was conducted to clarify the relationship between MAFLD and CAS.

## Methods

**Figure 1:**
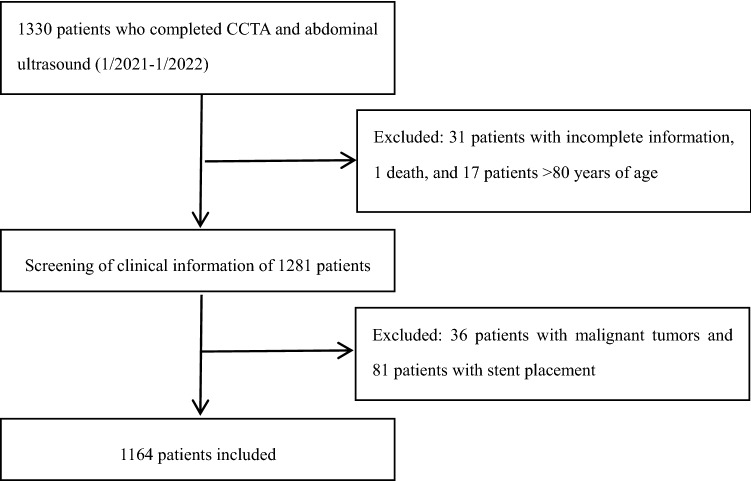
Flowchart for the inclusion of study subjects.

This study retrospectively included patients who underwent both coronary computed tomography angiography (CCTA) and abdominal ultrasound during their stay at the Second Affiliated Hospital of Kunming Medical University from January 2021 to January 2022. The exclusion criteria were as follows: (1) patients aged < 18 or > 80 years; (2) patients who had been discharged from the hospital or had a clear diagnosis of malignant tumors; (3) patients who had undergone coronary stent implantation or coronary artery bypass grafting; and (4) patients who died in the hospital. The study population was divided into an MAFLD group and a non-MAFLD group using the diagnostic criteria for MAFLD. The diagnostic criteria for MAFLD are based on histology, imaging or blood biomarker evidence of fat accumulation in the liver (hepatic steatosis) and one of the following three criteria: overweight/obesity [body mass index (BMI) > 23 kg/m^2^], type 2 diabetes mellitus (T2DM), or evidence of metabolic dysfunction. Metabolic dysfunction was defined as the presence of at least two of the following risk factors for metabolic abnormalities: (1) waist circumference ≥ 90 cm in men and ≥ 80 cm in women; (2) blood pressure (BP) ≥ 130/85 mmHg or receiving specific drug treatment; (3) plasma triacylglycerol ≥ 1.70 mmol/L or receiving specific drug treatment; (4) plasma high-density lipoprotein cholesterol (HDL-C) < 1.0 mmol/L in males and < 1.3 mmol/L in females or receiving specific drug treatment; (5) prediabetes, i.e., fasting blood glucose (FBG), 5.6–6.9 mmol/L; 2-h postprandial blood glucose (PBG), 7.8–11.0 mmol/L; or glycosylated hemoglobin (HbA1c), 5.7–6.4%; (6) steady-state insulin resistance index ≥ 2.5; and (7) plasma high-sensitivity C-reactive protein (CRP) > 2 mg/L^5^. A total of 1164 patients were included in the study (Fig. 1). This retrospective study involving human participants met the ethical standards of the Ethics Committee of the Second Affiliated Hospital of Kunming Medical University and the 1964 (Helsingin Declaration) and its subsequent amendments.The need for informed consent was waived by the Ethics Committee of the Second Affiliated Hospital of Kunming Medical University.

### Clinical and experimental data

The participants’ basic demographic data were obtained from a database at the Second Affiliated Hospital of Kunming Medical University. Laboratory indicators, sex, age, BMI, medication history, smoking history, and chronic disease history (hypertension and type 2 diabetes) were collected. Laboratory indicators included complete blood count, aspartate aminotransferase (AST), alanine aminotransferase (ALT), triglyceride (TG), high-density lipoprotein (HDL-C), low-density lipoprotein cholesterol (LDL-C), blood urea, creatinine, uric acid, FBG, and HbA1c. Obesity was defined as BMI ≥ 28.0 kg/m^2^^9^. Diabetes was defined as FBG concentration ≥ 126 mg/dL, self-reported history of diabetes, or the use of antidiabetes drugs. Hypertension was defined as BP ≥ 140/90 mmHg, self-reported history of hypertension or diagnosis by a doctor during hospitalization^10^^.^ Dyslipidemia was defined as elevated serum cholesterol and/or TG, commonly known as hyperlipidemia (total cholesterol (TC) ≥ 5.2 mmol/L, TG ≥ 1.7 mmol/L, LDL-C ≥ 3.4 mmol/L, or HDL-C < 1.0 mmol/L).

### CCTA image acquisition and analysis

CCTA scans of the coronary arteries were performed using a Canon Aquilion One 320-slice CT scanner (resolution, 1024 × 1024). Using prospective electrocardiogram (ECG)-triggered acquisition, 4 × 2.5 mm slices were collimated at 100 kV and 120 mAs at 50% of the RR interval, and images were acquired in 3.0-mm slices for the entire coronary artery area. A set of sequences with the best coronary angiography were selected and imported into a workstation (CoronaryDoc, Shukun Technology, Beijing, China). All CCTA scans were analyzed by experienced cardiovascular radiologists. CCTA analysis was conducted in accordance with the guidelines of the Society of Cardiovascular Computed Tomography to assess plaque type and degree of coronary artery stenosis^11^. The degree of coronary artery stenosis was graded as follows: slight stenosis, 1–29%; mild stenosis, 30–49%; moderate stenosis, 50–69%; severe stenosis, 70–99%; and occlusion, 100%^12^. Plaques occupied by calcified tissue covering more than 50% of the plaque area (density > 130 Hounsfield units in native scans) were classified as calcified plaques, plaques with < 50% calcium were classified as mixed plaques, and plaques without calcium were classified as noncalcified plaques^13^. One coronary artery stenosis ≥ 50% was defined as a single lesion, and stenosis ≥ 50% in ≥ 2 vessels was defined as multiple lesions.

### Ultrasonography

Fatty liver was confirmed by ultrasound using a Philips IU Elite color Doppler ultrasound system. A professional ultrasound physician performed B-mode ultrasound examinations of the liver of each participant. Fatty liver was diagnosed if one of the following criteria was met: (1) enhanced echo signals in the near-field of the liver, (2) unclear structure of the intrahepatic ducts, and (3) weakened echo signals in the far-field of the liver^14^.

### Statistical analysis

Continuous data are expressed as the mean ± standard deviation (SD). To compare data between groups, the independent samples t test was used for continuous variables, Fisher’s exact test was used for categorical variables, and the Wilcoxon rank sum test was used for ordinal variables. To analyze the relationships of MAFLD with coronary atherosclerotic plaques and significant coronary artery stenosis on CCTA, univariate and multivariate logistic regression analyses were performed. The covariates in the multivariate model (age, sex, smoking, BMI, hypertension, diabetes, and LDL) were selected based on clinical importance and statistical significance. *p* < 0.05 was considered statistically significant. SPSS software (version 25.0) was used for data processing and statistical analyses.

### Ethical approval

The study was performed according to the guidelines of the Helsinki Declaration and was approved by the Ethics Committee of the Second Affiliated Hospital of Kunming Medical University. Ethical Approval Number: Trial-PJ-project-2022-106.

## Results

The clinical medical records were reviewed, and patients with incomplete data, patients who died during hospitalization, patients with malignant tumors, patients with coronary stent placement before or during hospitalization, and patients < 18 years of age or > 80 years of age were excluded. Ultimately, 1164 patients were included in the study. Using the MAFLD diagnostic criteria, 680 patients (58.41%) were enrolled in the MAFLD group, and 484 patients (41.59%) were enrolled in the non-MAFLD group. The baseline characteristics of the patients in the MAFLD and non-MAFLD groups are shown in Table 1. The average age of the study population was 60.68 ± 10.32 years, and 606 (52%) participants were male. There were more male MAFLD patients than female MAFLD patients. Compared with patients in the non-MAFLD group, patients in the MAFLD group had higher BP, BMI, TGs, and FBG. HDL levels were lower in the MAFLD group than in the non-MAFLD group. Hypertension, diabetes, and hyperlipidemia were more common in the MAFLD group than in the non-MAFLD group. The percentages of patients with a history of smoking was similar between the MAFLD group and the non-MAFLD group, consistent with previous studies^12^.

**Table 1:** Baseline characteristics of the MAFLD group and non-MAFLD group.

Characteristic	Total (n = 1164)	MAFLD (n = 680)	Non-MAFLD (n = 484)	t/z/χ^2^	*P*
Male	606 (52%)	363 (53.38%)	243 (50.20%)	1.14	0.280
Age	60.68 ± 10.32	60.77 ± 10.51	60.56 ± 10.05	0.34	0.710
BMI	24.92 ± 3.87	26.30 ± 3.59	22.98 ± 3.38	15.92	**0.000**
Systolic BP	126.90 ± 16.19	129.28 ± 16.06	123.57 ± 15.82	0.84	**0.000**
Diastolic BP	78.04 ± 11.31	79.38 ± 11.21	76.17 ± 11.20	0.79	**0.000**
Type 2 diabetes	345 (29.64%)	258 (37.94%)	87 (12.79%)	54.05	**0.000**
Hypertension	732 (62.80)	487 (71.62%)	245 (50.41%)	53.42	**0.000**
Smoking	324 (27.84%)	196 (28.82%)	128 (26.45%)	16.05	0.372
Obesity	214 (18.38%)	183 (26.75%)	31 (6.4%)	79.24	**0.000**
Lipid-lowering drugs	894 (76.80%)	553 (81.32%)	341 (70.45%)	18.75	**0.000**
Hyperlipidemia	674 (57.90%)	465 (68.38%)	275 (56.81%)	16.33	**0.000**
TG	2.04 ± 1.79	2.41 ± 2.13	1.51 ± 0.93	-11.76	**0.000**
HDL	1.13 ± 0.30	1.08 ± 0.26	1.20 ± 0.33	-6.36	**0.000**
LDL	2.90 ± 0.95	2.93 ± 0.92	2.86 ± 0.88	-3.21	0.158
FBG	6.18 ± 2.39	6.48 ± 2.47	5.76 ± 2.20	-7.33	**0.000**
ALT	26.71 ± 21.96	28.77 ± 22.20	23.82 ± 21.31	-7.52	**0.000**
AST	25.63 ± 25.75	25.55 ± 24.16	25.74 ± 27.84	-1.11	0.900
Creatinine	75.04 ± 31.47	74.95 ± 28.98	75.17 ± 34.69	-1.01	0.314
Urea	5.68 ± 2.50	5.53 ± 1.88	5.90 ± 3.16	-1.26	0.207
Uric acid	385.44 ± 106.02	405.60 ± 98.42	357.11 ± 109.87	-7.98	**0.000**

*BMI* Body mass index; *TG* Total cholesterol; *HDL* High-density lipoprotein; *LDL* low-density lipoprotein; *FBG* fasting blood glucose; *ALT* alanine aminotransferase; *AST* aspartate aminotransferase.

Significant values are in [bold].

Table 2 lists the types and severity of atherosclerotic plaques in coronary arteries. CAS was found in 992 (79.20%) of the 1164 patients. In the MAFLD group, 81.32% of patients had CAS (defined as any plaque in the coronary arteries), and in the non-MAFLD group, 76.23% of patients had CAS; the difference was not statistically significant. Calcified plaques, noncalcified plaques, and mixed plaques were detected in 508 (43.64%), 206 (17.70%), and 286 (24.57%) patients, respectively. The prevalence of calcified plaques, noncalcified plaques, and mixed plaques each was significantly higher in MAFLD patients than in non-MAFLD patients (*p* < 0.05). A total of 302 patients (25.95%) had significant coronary artery stenosis (stenosis ≥ 50% diameter), and the degree of significant coronary artery stenosis was significantly higher in the MAFLD group than in the non-MAFLD group. There was no significant difference between the MAFLD and non-MAFLD groups with regard to single vascular lesions (one coronary artery stenosis ≥ 50%), but multiple vascular lesions was significantly more prevalent in the MAFLD group than in the non-MAFLD group. Among all the participants, significant stenosis of the right coronary artery, left main stem, left anterior descending branch, and left circumflex coronary artery was observed in 127 (10.91%), 22 (1.89%), 229 (19.67%), and 76 (6.5%) participants, respectively.

**Table 2:** Comparison of CCTA results between MAFLD patients and non-MAFLD patients.

Characteristic	Total	MAFLD	Non-MAFLD	χ^2^	*P*
Overall plaque	922 (79.20%)	553 (81.32%)	369 (76.23%)	0.74	0.338
Calcified plaques	508 (43.64%)	320 (47.05%)	188 (38.84%)	7.76	**0.005**
Noncalcified plaques	206 (17.70%)	144 (21.18%)	62 (12.81%)	13.58	**0.000**
Mixed spots	286 (24.57%)	186 (27.35%)	100 (20.66%)	6.83	**0.009**
No stenosis	185 (15.89%)	101 (14.85%)	84 (17.36%)	1.32	**0.034**
Nonobstructive stenosis (1–49%)	677 (58.61%)	386 (56.76%)	291 (60.12%)	1.31	**0.034**
Obstructive stenosis (≥ 50%)	302 (25.95%)	193 (28.38%)	109 (22.52%)	5.06	**0.034**
Single vascular lesion (≥ 50%)	194 (16.67%)	113 (16.62%)	81 (16.74%)	0.00	0.958
Multiple vascular lesions (≥ 50%)	108 (9.28%)	80 (16.53%)	28 (5.78%)	12.01	**0.004**

Significant values are in [bold].

The correlation between MAFLD and CAS is detailed in Tables 3, 4, 5. In the univariate logistic regression, MAFLD was significantly correlated with overall plaque, calcified plaques, noncalcified plaques, and mixed plaques in the coronary arteries. MAFLD was also significantly correlated with significant coronary artery stenosis (*p* < 0.05). However, after correction for cardiovascular risk factors (age, sex, smoking history, BMI, diabetes, hypertension, and hyperlipidemia ), logistic regression analysis indicated that MAFLD was not correlated with overall plaque (1.15, 95% confidence interval [CI]:0.82–1.62; *p* = 0.396), calculated plaques (1.28, 95% CI: 0.97–1.69; *p* = 0.076), or significant stenosis (1.00, 95% CI: 0.73–1.37; *p* = 0.979). In contrast, MAFLD was significantly correlated with noncalcified plaques (1.64, 95% CI: 1.14–2.35; *p* = 0.007) and mixed plaques (1.46, 95% CI: 1.06–2.01; *p* = 0.020).

**Table 3:** Univariate logistic regression analysis of coronary artery stenosis and plaque with MAFLD and risk factors.

Characteristic	Significant stenosis			Calcified plaques			Overall plaque		
	OR	95% CI	*P*	OR	95% CI	P	OR	95% CI	*P*
MAFLD	1.36	1.04–1.79	**0.025**	1.42	1.12–1.80	**0.004**	1.35	1.02–1.80	**0.036**
Male	1.40	1.08–1.83	**0.012**	1.16	0.91–1.46	0.213	1.34	1.01–1.78	**0.043**
Age	1.04	1.03–1.06	**0.000**	1.05	1.03–1.06	**0.000**	1.05	1.04–1.07	**0.000**
Smoking	1.25	0.95–1.65	0.106	1.20	0.92–1.55	0.162	1.30	0.95–1.77	0.097
BMI	1.03	0.99–1.06	0.117	1.01	0.98–1.04	0.422	1.02	0.98–1.06	0.249
Hypertension	1.80	1.35–2.39	**0.000**	1.32	1.03–1.68	**0.023**	1.88	1.41–2.50	**0.000**
Type 2 diabetes	2.83	2.15–3.72	**0.003**	1.77	1.37–2.28	**0.000**	1.65	1.18–2.31	**0.003**
Hyperlipidemia	0.94	0.72–1.24	**0.667**	1.16	0.91–1.48	0.217	1.24	0.93–1.66	**0.140**
TG	1.00	0.94–1.08	0.814	0.97	0.90–1.04	0.408	0.99	0.90–1.05	0.537
HDL	0.54	0.34–0.85	**0.008**	0.75	0.51–1.11	0.158	0.65	0.40–1.02	0.061
FPG	1.12	1.07–1.18	**0.000**	1.06	1.01–1.12	**0.008**	1.08	1.01–1.17	**0.021**

Significant values are in [bold].

**Table 4:** Univariate logistic regression analysis of coronary artery plaque with MAFLD and risk factors.

Characteristic	Noncalcified plaques			Mixed plaques		
	OR	95% CI	*P*	OR	95% CI	*P*
MAFLD	1.83	1.32–2.52	**0.000**	1.42	1.08–1.88	**0.012**
Male	1.12	0.82–1.51	0.465	1.74	1.32–2.29	**0.000**
Age	0.99	0.98–1.00	0.464	1.03	1.02–1.04	**0.000**
Smoking	1.06	0.76–1.45	0.739	1.05	0.79–1.39	0.737
BMI	1.05	1.00–1.08	**0.018**	0.98	0.94–1.01	0.308
Hypertension	1.46	1.05–2.02	**0.022**	1.39	1.05–1.85	**0.020**
Type 2 diabetes	1.35	0.98–1.85	0.066	1.99	1.51–2.64	**0.000**
Hyperlipidemia	0.81	0.60–1.12	0.204	0.78	0.59–1.02	0.079
TG	0.99	0.91–1.08	0.914	0.99	0.92–1.07	0.962
HDL	0.69	0.41–1.15	0.162	0.80	0.51–1.26	0.344
FPG	1.07	1.01–1.13	**0.013**	1.07	1.01–1.12	**0.009**

Significant values are in [bold].

**Table 5:** Multivariable regression analysis of coronary artery stenosis and plaque pattern with MAFLD severity and risk factors.

	Overall plaque		Calcified plaques		Noncalcified plaques		Mixed plaques		Significant stenosis	
	OR (95% CI) CI)	*P*	OR (95% CI)	*P*	OR (95% CI)	*P*	OR (95% CI)	*P*	OR (95% CI)	*P*
CRUDE										
Non–MAFLD	1 (reference)	–	1 (reference)	–	1 (reference)	–	1 (reference)	–	1 (reference)	–
MAFLD	1.35 (1.02–1.80)	0.036	1.42 (1.12–1.80)	0.004	1.82 (1.32–2.52)	0.000	1.42 (1.08–1.88)	0.012	1.36 (1.04–1.78)	0.025
Model 1										
Non–MAFLD	1 (reference)	–	1 (reference)	–	1 (reference)	–	1 (reference)	–	1 (reference)	–
MAFLD	1.34 (1.00–1.80)	0.049	1.41 (1.10–1.81)	0.005	1.82 (1.31–2.52)	0.000	1.40 (1.06–1.87)	0.017	1.34 (1.01–1.77)	0.037
Model 2										
Non–MAFLD	1 (reference)	–	1 (reference)	–	1 (reference)	–	1 (reference)	–	1 (reference)	–
MAFLD	1.21 (0.87–1.69)	0.237	1.36 (1.04–1.79)	0.024	1.72 (1.21–2.46)	0.003	1.63 (1.19–2.24)	0.002	1.23 (0.90–1.67)	0.180
Model 3										
Non–MAFLD	1 (reference)	–	1 (reference)	–	1 (reference)	–	1 (reference)	–	1 (reference)	–
MAFLD	1.16 (0.83–1.61)	0.379	1.35 (1.03–1.77)	0.029	1.67 (1.17–2.39)	0.004	1.59 (1.16–2.18)	0.004	1.17 (0.86–1.60)	0.293
Model 4										
Non–MAFLD	1 (reference)	–	1 (reference)	–	1 (reference)	–	1 (reference)	–	1 (reference)	–
MAFLD	1.13 (0.80–1.58)	0.474	1.28 (0.97–1.68)	0.079	1.63 (1.13–2.33)	0.008	1.45 (1.06–2.02)	0.020	1.01 (0.74–1.39)	0.922
Model 5										
Non–MAFLD	1 (reference)	–	1 (reference)	–	1 (reference)	–	1 (reference)	–	1 (reference)	–
MAFLD	1.12 (0.80–1.57)	0.511	1.26 (0.96–1.67)	0.101	1.66 (1.16–2.38)	0.006	1.50 (1.08–2.06)	0.014	1.01 (0.74–1.39)	0.937

Model 1: sex, age, smoking.

Model 2: sex, age, smoking, BMI.

Model 3: sex, age, smoking, BMI, hypertension.

Model 4: sex, age, smoking, BMI, hypertension, Type 2 diabetes.

Model 5: sex, age, smoking, BMI, hypertension, Type 2 diabetes, Hyperlipidemia.

## Discussion

This study confirmed that MAFLD group had more cardiovascular risk factors ,

MAFLD is correlated coronary atherosclerotic plaques and significant stenosis. In addition, after correction for cardiovascular risk factors, MAFLD was independently correlated with the number of noncalcified plaques and mixed plaques, indicating that there is a correlation between MAFLD and CAS.

MAFLD group had more cardiovascular risk factors ,such as hypertension, diabetes, obesity, and dyslipidemia, which are common risk factors for CVD. Previous studies have found NAFLD to be correlated with CVD. A study involving 17,350 patients^15^ found that NAFLD was correlated with an elevated 10-year risk of developing CVD as estimated using the Framingham risk score (FRS) and was not correlated with classical CVD risk factors or MetS. Another study of patients with fatty liver with an average follow-up of 6.5 years found that this group’s risk of CVD doubled^16^. Since NAFLD was renamed MAFLD, hypertension, diabetes, obesity, and dyslipidemia have been included in its diagnostic criteria. Therefore, the relationship between MAFLD and CVD is closer than that between NAFLD and CVD. The complex mechanism linking NAFLD and CVD has not been fully elucidated, but inflammation is an important link; the mechanism that links MAFLD and CVD must be further studied.

This study found that MAFLD was correlated with overall plaque in the coronary arteries. After exclusion of recognized risk factors for coronary heart disease, such as age, sex, hypertension, diabetes, smoking, and hyperlipidemia, MAFLD was still independently correlated with the numbers of noncalcified plaques and mixed plaques. Niikura et al.^17^ found that NAFLD was closely correlated with increased carotid intima–media thickness, CAS, and increased arterial stiffness. A meta-analysis of 27 studies indicated that after excluding traditional cardiovascular risk factors and MetS, nonalcoholic fatty liver and subclinical AS were still independently correlated^18^. The latest evidence suggests that NAFLD is a risk factor for the development of cardiovascular complications of AS, such as stroke and myocardial infarction^19–21^. AS is one of the causes of CVD, and inflammation plays an important role in intimal thickening of the arterial wall, arteriosclerosis, and luminal stenosis^22^. An increase in CRP in fatty liver patients indicates an inflammatory process^23^, but the underlying mechanism is more complicated and may be related to chronic fat overload-induced liver cell death in NAFLD. Hepatocyte death causes the release of molecules that trigger macrophage activation, and an increase in macrophages and Kupffer cells leads to an increase in the circulating levels of systemic inflammatory markers, including the interleukin-1, -6, and -20 subfamilies^24,25^. MAFLD patients should be screened for atherosclerotic cardiovascular disease (ASCVD).

Most studies of NAFLD and CAS have suggested that NAFLD is only correlated with noncalcified plaques or mixed plaques^26,27^, but the results of this study suggest that MAFLD is correlated with both noncalcified and mixed plaques, indicating that MAFLD is more closely correlated with CVD than NAFLD. This closer correlation may be because the diagnostic criteria for MAFLD better identify lean or normal-weight fatty liver patients with metabolic abnormalities, a population that is not identified by the diagnostic criteria for NAFLD but is at high risk of developing CVD. A recent study revealed that MAFLD patients had a higher risk of ASCVD than did NAFLD patients^28^, and a retrospective study of 13,083 patients with complete ultrasound and laboratory data demonstrated that patients who met the diagnostic criteria for MAFLD were more likely to have multiple metabolic comorbidities, including CVD, than were patients with NAFLD^29^. Therefore, clinicians should use the diagnostic criteria for MAFLD to identify patients with metabolic dysfunction at an early stage and intervene promptly to reduce the incidence of CVD.

According to Table 2, the MAFLD group had 144 noncalcified plaques and the non-MAFLD group had 62, the number of mixed-type plaques was 186 in the MAFLD group and 100 in the non-MAFLD group.the MAFLD group had 2.3 times as many noncalcified plaques and 1.86 times as many mixed-type plaques as the non-MAFLD group.A prospective study with 3 years of follow-up^30^ found that the probability of major cardiovascular events within 3 years was significantly higher in patients with noncalcified plaques and mixed plaques than in patients with calcified plaques (23%, 38%, and 6%), indicating that the prognosis of patients with noncalcified plaques and mixed plaques is poor. Mixed plaques include both calcified and noncalcified plaques. Noncalcified plaques are unstable plaques, and unstable plaque rupture predisposes individuals to a greater risk of acute coronary syndrome events^30^. Patients with MAFLD combined with CAS may have a higher risk of major cardiovascular events and acute coronary syndrome events.

This study had significantly more patients with multiple coronary artery stenoses in the MAFLD group than in the non-MAFLD group. Multiple coronary artery stenoses may lead to more adverse cardiovascular events and poor patient prognosis. Therefore, MAFLD patients should be screened for ASCVD. Patients with CAS should be screened for MAFLD to improve their metabolic dysfunction quickly to avoid adverse cardiovascular events.

This study’s first limitation was that it could not clarify the causal relationship between MAFLD and coronary plaque formation. Second, because image reconstruction artifacts associated with radioactive materials (such as calcium metal) can obscure the coronary artery lumen, such artifacts may lead to an underestimation or overestimation of coronary artery stenosis (inaccurate assessment of the vascular lumen under high-calcium conditions). Third, the sample size of this study was small, and the findings were conservative. Even after correcting for age, sex, smoking, BMI, hypertension, and diabetes, which are recognized potential risk factors for coronary atherosclerosis in this study, there may still be some residual risk factors, and those remaining potential risk factors that were not corrected for may have impacted the study results. Therefore, we did not have sufficient power to rule out more subtle associations, for which a larger study is necessary. Finally, since this was a retrospective study, it was not possible to trace the specific time a medication was started and the liver condition before it was started, such as antihypertensive drugs, lipid-lowering drugs, hypoglycemic drugs, and weight-loss drugs, which have better preventive and therapeutic effects on diseases such as CVD and metabolic dysfunction and therefore may have caused an underestimation of the actual prevalence of MAFLD and CVD.

In summary, this retrospective study found that MAFLD had more cardiovascular risk factors, MAFLD was correlated with coronary atherosclerosis,and significant stenosis.Further study found independent associations between MAFLD and the numbers of noncalcified plaques and mixed plaques, which suggest a clinically important link between MAFLD and coronary atherosclerosis.

## Data Availability

The datasets used and/or analyzed during the current study are available from the corresponding author on reasonable request.

## Abbreviations

NAFLD: Nonalcoholic fatty liver disease
MetS: Metabolic syndrome
CAS: Coronary atherosclerosis
MAFLD: Metabolic-associated fatty liver disease
CCTA: Coronary computed tomography angiography
NASH: Nonalcoholic steatohepatitis
AS: Atherosclerosis
CVD: Cardiovascular disease
BMI: Body mass index
BP: Blood pressure
TG: Total cholesterol
TC: Total cholesterol
HDL: High-density lipoprotein
LDL: Low-density lipoprotein
FBG: Fasting blood glucose
PBG: Postprandial blood glucose
HbA1c: Glycosylated hemoglobin
CRP: C-reactive protein
AST: Aspartate aminotransferase
ALT: Alanine aminotransferase
ASCVD: Atherosclerotic cardiovascular disease
